# Context-based learning for Inhibition of alternative conceptions: the next step forward in science education

**DOI:** 10.1038/s41539-018-0026-9

**Published:** 2018-06-22

**Authors:** Alexandra Renouard, Yves Mazabraud

**Affiliations:** 1 Université de Strasbourg, CNRS, IPGS, UMR 7516, 5 rue René Descartes, 67100 Strasbourg, France; 2 Université des Antilles, Géosciences Montpellier UMR 5243, Fouillole, 97159 Pointe à Pitre, France; 3 Université des Antilles, Ecole Supérieure du Professorat et de l’Education de Guadeloupe, CRREF EA 4538, Morne Ferret, 97178 Abymes, France

## Abstract

The scientific literacy level of the whole population has long been focusing the researchers’ attention because of its direct impact on many aspects of our lives. As a matter of fact, studies in cognition have both been inspired by educational issues as well as by misconceptions of scientific ideas often based on irrational beliefs, old theories, unscientific reasoning, or unassimilated conceptual instruction. As a result, individual conceptions are now accurately described in many scientific fields, which has led to improvements in science teaching and learning. However, the community (scientists, academics, high school and primary school teachers, and educators) has not yet succeeded in solving all the issues, so some pre-existing misconceptions still persist in the population. In this paper, we argue that cognition studies must now focus on the origin of individuals’ conceptions and on their modes of acquisition and propagation. The goal is to provide educational tools for acting upstream, during early scientific instruction, before the very acquisition of scientific conceptions.

## Introduction

Twentieth-first century challenges often remind us how essential the understanding of science is to mankind (global warming, natural disasters, energy and water access, personal health, epidemics, and pandemics). Many of our daily decisions—choosing domestic energy or a car, the most suitable living location in seismic, volcanic and flood zones, personal diet and physical activity, or following a medical treatment—are related to scientific notions. For instance, a more efficient health care can be promoted by involving the patients in medical decision-making. This allows better treatment adherence and reduces anxiety. Well-calibrated instruction can also prevent decline in vaccine coverage rates and help to protect the population.^1^ From the individual to the societal level, the more the decisions are scientifically supported, the greater the benefit. That’s why, to make the right choices, the general public, not only the elite scientific community, needs to be scientifically literate. Indeed, the interest for science education research has been growning worldwide in many fields: the creation of dedicated groups,^2,3^ structuring methodology,^4–6^ and clear definition of common objectives.^7^ Moreover, international syllabus of basic scientific concepts and skills are proposed to encourage global science literacy and more effective training for future professionnals.^8^ Finally, a substantial international study effort on conceptions developed by individuals is being widely deployed.^9–16^

Nevertheless, if everybody is daily exposed in the media to many scientific topics, not everyone has mastered the resulting concepts^17–19^, especially since scientific questions are embedded in a complex social and political context.^20,21^ Thus, when individuals’ conceptions are far from scientific ones (i.e., those generally accepted by the scientific community), choices and actions can have major consequences in everyday life.^22^ The seismic disaster that occurred in 2010 near Port-au-Prince, the capital of Haiti, is a good example. The high seismic hazard was well known by scientific experts, but, because of cognitive barriers, they did not succeed in raising awareness among decision makers. Indeed, the precise time of occurrence of an earthquake remains impossible to predict. Furthermore, their recurrence interval can be longer than a human lifespan. Before 2010, the last Haitian strong earthquake occurred in 1887, and no major event had taken place near Port-au-Prince, since 1770. Losing memory of a crisis, at the individual or societal level, makes it difficult for a related risk culture to develop, especially if the population is not familiar with the relevant scientific concepts (earthquakes periodicity at plate boundaries in that case). Seismic risk mitigation in such geological context therefore requires long-term policy (building codes, education, training, and spatial planning). Conversely, some other natural threats have a weaker intensity but are seasonally recurrent. The time scales between the probabilities of occurrence of different natural hazards and their maximum potential magnitude call for mathematical assessments (in particular, probabilities and logarithmic scale laws).

Without the proper scientific knowledge, a risk of periodic moderate-intensity event (like damaging floods) can be perceived as more dangerous than a risk of episodic high intensity event (like strong magnitude earthquakes), leading to a lack of preparation and poor crisis management. In Haiti, despite the seismologists’ warnings, governmental efforts for natural risks mitigation had been focused on repetitive and predictable threats (hurricanes, floods), leaving the country and the population unprepared for a seismic catastrophe.^23^

The correct scientific information, although available—and even provided—is then not always considered in the decision-making process. Instead, simplistic answers to complicated problems are accepted without really differentiating scientific explanations from nonscientific explanations.^17,22,24^ Yet, population resilience depends on a good perception of risk (natural disasters, health, drug-resistant bacteria, chemical incidents, pesticide residues in food, climate change, and shale gas exploitation…) which conditions safety behaviors.^18,25–27^

However, scientifically well-instructed people do not necessary make the best individual choices for the welfare and protection of society. For example, parents with high functional, communicative, and critical health literacy are paradoxically more likely not to vaccinate their children.^28^

So, despite the will to make each citizen familiar with science since the late 1950s,^29,30^ true science literacy remains difficult to achieve and individual conceptions hard to change.^19,30–33^

To mitigate the vulnerability of society to misunderstandings, misinterpretations, or misinformation, without oversimplifying the complex or overcomplicating the simple, it appears important to study the parameters that influence the individual decision-making processes and impede a meaningful scientific literacy. These factors are to be found in the specific context of each person. As a matter of fact, everyone interacts with his environment, and the links one weaves daily with each element of it influence his own way of seeing, thinking and understanding the world around him. These links, being influential, are then referred as interactions.

Recent research in neuroscience has shown that all the information taken from the environment is processed at the neural circuitry level. Relative decisions are then made based on a selective inhibitory control that enables or prevents the acquisition of scientific conceptions. As a consequence, when exposed to new and crucial information, individuals can interpret it with their own pre-existing conceptual framework, and this information is likely to reinforce the pre-existing conceptions, be rejected or be questioned.^34^

Context dependent interactions appear therefore to have an important impact on the learning process. That is why, to take a step forward, in this article we propose the principles of a synthetic learning model based on these interactions in the sole objective of improving science literacy. Quite apart from the classical transformist tradition of conceptual change, this approach focuses on the learning context rather than on conceptions themselves. The purpose is to act on brain inhibition processes by identifying precisely the individuals’ environmental factors that influence their conceptions and impede scientific learning.

## Learning without changing our conceptions?

Conceptions we have developed from our daily experiences form an effective cognitive processing system to help us think and react quickly, avoid threats, and value the ideas of our groups in many situations in our real lives.^35^ However, in light of scientific understanding, a recent phenomenon in human evolution,^29,36^ these kinds of conceptions can create real obstacles. In this case, they need to be replaced with more scientific conceptions that agree with up-to-date scientific findings. But, it is known since a long time that people do not overcome easily these cognitive obstacles, even when faced with new information.^37,38^

As a matter of fact, when this requires simple additions to knowledge or enrichment to already existing knowledge, overcoming erroneous conceptions is easy. But if a conflict emerges between the learned and the to-be-learned concepts, then it involves a radical conceptual change.^39^ In this process, all our conceptions that depart from scientific ones become misconceptions that need replacing, and cognitive conflict feeds this change.^40^

As a major challenge, study of conceptual change, of which theory is sometimes cited as an authority argument, self-justified by its famous historical foundations,^41^ has become an international necessity and results in several learning models.^42,43^ Nonetheless, activating direct cognitive conflict to learn new scientific concepts is controversial.^44^ Especially because individual conceptions, automated and well-established, are resistant to change, even after instruction, and persist as distractions against counter-intuitive scientific conceptions.^45^ Historical view of changing radically conceptions through classical instruction tends to show limits in efficiency, calling for a learning paradigm shift.

We then use the term “alternative conceptions”^46^ rather than “misconceptions”, since the aim is not to replace them with scientific ones but to refine them. Hence, acquisition of scientific conceptions results in increased inclinations that lead to correct scientific answers and scientific reasoning. This process controls the spontaneous tendency to use our original conceptions. Using functional magnetic resonance imaging, the existence of such a regulation has actually been demonstrated in neuroscience through the imaging of active brain areas in the prefrontal and anterior cingulate cortex.^45,47^ These areas are effectively associated with an inhibitory control of alternative conceptions.^48^ When activated during a given learning situation, they control other posterior brain regions that are associated with previous (i.e., alternative) conceptions and inhibit their activation.^49^ Inhibited, the pre-existing conceptual framework does not interfere with new data and learning is made possible. However, overcoming the conflict between new data and previous alternative conceptions is a demanding and time-consuming process. It depends on the efficiency of inhibitory control mechanisms and especially the individual level of expertize.^49^ As a matter of fact, when such conflict is unresolved, the brain areas associated with error detection, conflict monitoring, effortful processing and working memory tend to show increased level of activity.^45^ In that case, the new data is likely rejected, learning does not easily occur, conceptual change appears difficult to achieve and the alternative conceptions persist.

Therefore, the individual conceptual system is a conflictual, but dynamic and evolving system, marked by the coexistence of scientific conceptions and persisting alternative conceptions, more or less inhibited according to the individual’s expertize.^50^ Similar ideas have also been evocated by radical constructivism theoricists^51^ and, then, largely extended by neuroconstructivists.^52^

The educational challenge then lies in the design of the adequate teaching and learning scenarios accordingly, in order to avoid investing considerable time reteaching concepts that are not mastered even after instruction.^53–56^

## Taking a fresh look at our learning context

We all construct our own experience of the world in a specific context. This latter designates each element with which we interact specifically, such as the people around us, media, teaching and learning resources, or our living environment. These elements are named “contextual elements”. Thus, each individual selects the appropriate answers from his alternative and/or scientific conceptions that depend upon his specific context.

Within this specific context, people often attribute the origin of their alternative conceptions to school, but also to the media.^57^ It is clear that alternative conceptions can be conveyed in classrooms.^58^ Teachers may hold alternative conceptions in different areas: science content,^59^ science learning, students’ difficulties and conceptions,^60^ the nature of science.^61^ Thus, students’ alternative conceptions can sometimes echo their teacher’s understanding. To understand their teaching and to characterize its impact on each student in the classroom, it appears essential to describe the teacher’s contextual interacting. As a matter of fact, when teacher’s and student’s contexts are concomitantly known, their relative interactions are more apprehensible. The design of a specific learning and teaching environment, based upon targeted evolution of each context, is then possible.

Media resources are another context element to consider. Indeed, in an increasingly visual society, science fiction films, for example, which blur reality and fiction, can convey misunderstandings that alter the public’s critical analysis and corroborate common alternative conceptions.^62^ This can constitute a real obstacle to the development of citizens’ scientific literacy and accelerate the perpetuation of alternative conceptions.^22,24,63^ Studying the specific media habits of students is therefore essential. It helps us understand the nature and result of interactions that they maintain with the media resources they consult so we can act upon them.

In the same way, teaching and learning resources such as textbooks are another element of context to consider. Many studies show that textbooks convey and reinforce alternative conceptions in various scientific fields.^64–66^ Analyzing interactions between teaching or learning resources and students will yield extra data to better define each individual context.

In addition, every individual is necessarily inscribed within his everyday natural environment and can easily observe commonplaces, landscapes, weather, biotopes, and outcrops. Therefore, through interactions within this physical environment, each one experiments with the field by moving through it to reveal multiple perspectives. Their specific sensory-motor field experience determines their perceptions and their interpretations of what they observe.^67^ It is then obvious that studying the interactions between individuals and their living environment is also essential. It reveals other factors influencing alternative conceptions to act on.

Nonetheless, people are not only integrated into a physical environment, but also live and work together to see, understand, and represent the world in a social environment. Therefore, family members, friends or peer-to-peer interactions can convey metaphors, images of past experiences, epistemological commitments, metaphysical beliefs, and knowledge that are involved in the establishment of an individual’s alternative and/or scientific conceptions. Thus, social (people surrounding)^68,69^ and cultural (beliefs, languages)^70^ interactions are also fundamental to the emergence of alternative conceptions.

**Fig. 1:**
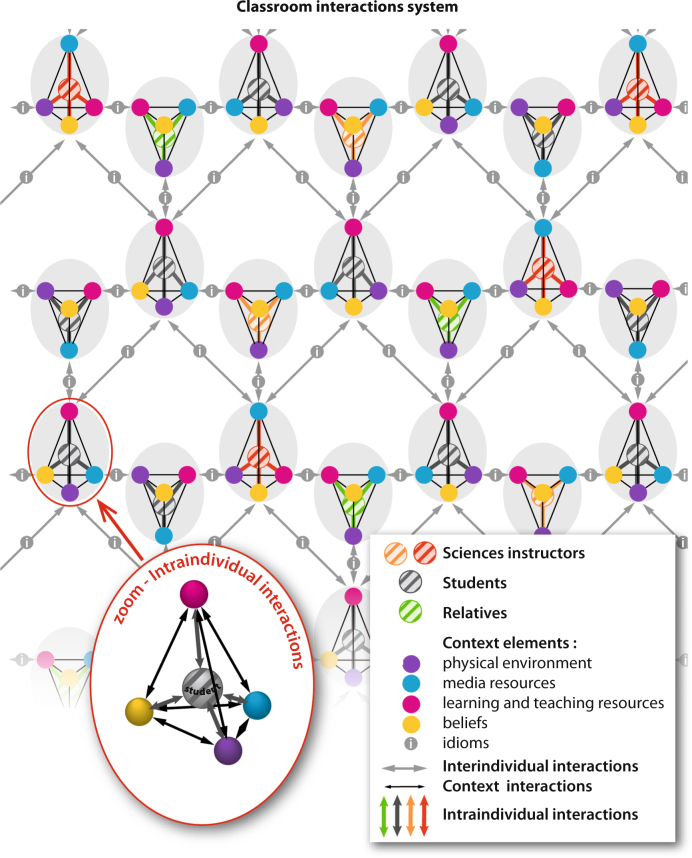
Individual and class learning model based on interactions

The contextual elements, associated with the intra- and the inter-individual interactions, as well as the idioms used for communication, constitute each individual context. Hence, the assembly of all the people collaborating with each other forms a dense nexus that constitutes the learning environment’s framework. When such environment is modeled, an intricately interconnected structure of individual contexts is effectively enlightened (Fig. 1). From the personal to the population level, this network of links becomes increasingly complex. Among them, the existence of parents to children and teachers to students’ connections support the idea of an intergenerational transmission of alternative conceptions.

## Inhibiting alternative conceptions by destabilizing the strongest interactions?

Interactions between individuals and contextual elements are then the preferred targets for any learning action. As they are at the heart of the system and structure all the frame, they appear essential for the limitation of alternative conceptions’ transmission. Indeed, impacting on them allows renewing the relationship between an individual and his surroundings, up to the scale of an entire society. However, as everyone interacts more or less deeply with his contextual elements, it is first necessary to identify the strongest interactions, namely the most stable and resistant, that hinder scientific conceptions acquisition. Because of human variance, not everyone will have the same kind of interactions. Parameters that define an interaction, and that may vary, are its nature (human-human, human-object, technological, verbal, peer-to-peer, or hierarchical relationship), as well as its intensity (relative importance in the construction of knowledge), its stability (associated with old and reinforced habits/knowledge versus new, sentimental value or emotional attachment, degree of trust and esteem of people involved) and its resistance (ability or reluctance to change under external forcing, whether conscious or unconscious). The relative quantification of these parameters, by specific criteria that need to be chosen for each study, allows discriminating strong interactions from weak.

**Fig. 2:**
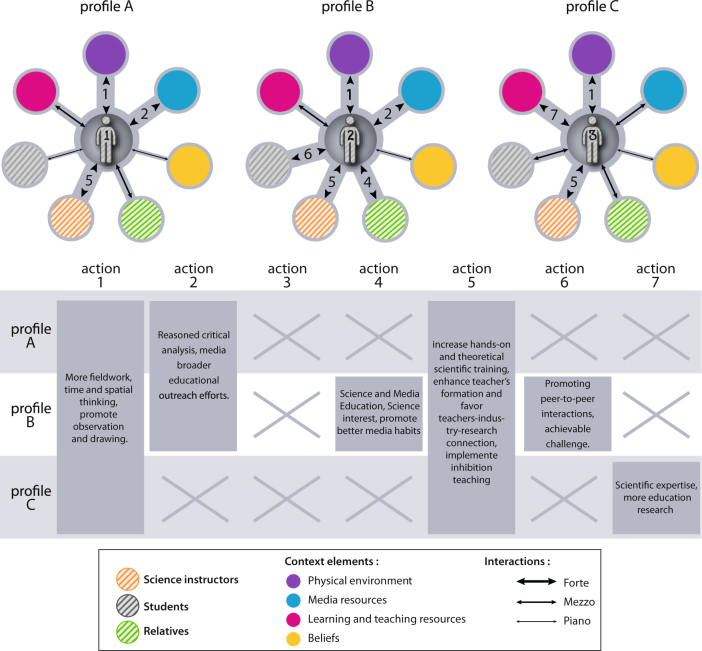
Examples of student’s profiles and possible targeted actions. The width of the link between the individual and the context elements reflects its robustness

Second, we aim to establish individual students’ type of profiles in the light of those strongest interactions. Finally, we seek to trigger the evolution of the interactions by designing a targeted scenario according to the students’ profiles. Rather than on the conceptions themselves, educational attention here consequently focusses on the interactions, like the student’s media habits or the teacher-student trust relationship or the involvement of parents in the schooling for example. As a matter of fact, the idea is to favor scientific learning by stimulating efficiently brain inhibitory processing. To do so, action strategies may be carried out to weaken specifically interactions that are considered to be blocking the inhibition procedure, and, to strengthen the ones that are promoting it (Fig. 2).

Such promising actions may be developed at various levels. A single teacher in his classroom may adapt his teaching to newly surveyed students, for example with more fieldwork or textbook critical analysis. Depending on their personal profiles, differentiated instruction can be easily designed and students assigned specific exercises or tasks in a group project. In a whole academic student pool, or at national level, the studies’ outcomes may be useful to university developers for adapting curricula to the local population. As a matter of fact, the number of identified types of student profiles (see examples in fig. 2) and their percentage within the whole studied population, as well as their repartition, define an atlas that may be taken into account to either change the curriculum or to design temporary pedagogical projects.

This kind of atlas requires either an entry for each individual or for a statistically representative group of individuals selected within the population under study.

A continuous census of individual profiles can constitute a rich database that would also allow studying educational discrepancies relative to other parameters such as the location of the schools or the socio-economical, cultural and linguistic environment of the students. Depending on the size of the population, the necessary data for establishing profiles can be collected by interviews or Multiple Choice Questions for instance. Large population shall be surveyed via online questionnaires, authorizing automated data processing and common profiles characterization. On the other side, small size sample studies can rather favor semi-guided interviews for the generation of individual profiles. In all cases, the dataset shall enable the quantification, in a chosen grid, of the interactions between the subject and the various parameters of his environment.

## Conclusion

Many common alternative conceptions in science are very present in the population, especially if the social environment of the individual outside the classroom is devoid of scientific knowledge. In this way, the study of synthetic learning models based on interactions should be conducted in early schooling to create the possibility of very early action and therefore a better chance of success. Indeed, under these conditions, alternative conceptions will be transmitted more rarely, implying that fewer alternative conceptions will be conveyed to the university and in adulthood. Nevertheless, as the interactions between students and contextual elements are evolving through time, this synthetic model can be established at any point of schooling and at different stages of the student’s life. It can be therefore readjusted and evolved by redefining finely targeted action strategies to apply.

Moreover, interaction-centered studies in favor of inhibition of alternative conceptions for more scientific ones, benefits not only to the students but to society in general. Among the individuals that we scientifically literate, there are future engineers, teachers, science journalists, writers of textbooks or other resources, decision makers, and future parents. So, in the light of interactions networks, all these people may positively influence, directly or indirectly, every other member of the society.

### Data availability statement

No datasets were generated or analyzed during the current study.
